# Lyn mediates FIP1L1-PDGFRA signal pathway facilitating IL-5RA intracellular signal through FIP1L1-PDGFRA/JAK2/Lyn/Akt network complex in CEL

**DOI:** 10.18632/oncotarget.11401

**Published:** 2016-08-19

**Authors:** Bin Li, Guangsen Zhang, Cui Li, Ruijuan Li, Jingchen Lu, Zhengxi He, Quan Wang, Zhenzi Peng, Jun Wang, Yeping Dong, Chunfang Zhang, Jie Qiong Tan, Nacef Bahri, Yuexiang Wang, Chaojun Duan

**Affiliations:** ^1^ Medical Research Center, Key Laboratory of Cancer Proteomics of Chinese Ministry of Health, Xiangya Hospital, Central South University, Changsha, People’s Republic of China; ^2^ Division of Hematology, Institute of Molecular Hematology, The Second Xiang Ya Hospital, Central South University, Changsha, People’s Republic of China; ^3^ Division of Oncology, Xiangya Hospital, Central South University, Changsha, People’s Republic of China; ^4^ State Key Laboratory of Medical Genetics, Xiangya Medical School, Central South University, Changsha, People’s Republic of China; ^5^ Department of Pathology, Brigham and Women's Hospital and Harvard Medical School, Boston, Massachusetts, USA; ^6^ The Institute of Health Sciences, Shanghai Institutes for Biological Sciences, Chinese Academy of Sciences/Shanghai Jiao Tong University School of Medicine, Shanghai, People’s Republic of China

**Keywords:** Lyn, CEL

## Abstract

The Fip1-like1 (FIP1L1)–platelet-derived growth factor receptor alpha (PDGFRA) (F/P) oncogene can cause chronic eosinophilic leukemia (CEL), but requires IL-5 cytokine participation. In this study, we investigate the mechanism of F/P in collaboration with IL-5 in CEL. The results showed that Lyn, a key effector in the IL-5-motivated eosinophil production, is extensively activated in F/P-positive CEL cells. Lyn can associate and phosphorylate IL-5 receptor α (IL-5RA) in F/P-positive cells. Moreover, the activation of Lyn and IL-5R kinase were strengthened when the cells were stimulated by IL-5. Lyn inhibition in F/P-positive CEL cells attenuated cellular proliferation, induced apoptosis, and blocked cell migration and major basic protein (MBP) release. We identified the FIP1L1-PDGFRA/JAK2/Lyn/Akt complex in the F/P-expressing cells which can be disrupted by dual inhibition of JAK2 and Lyn, repressing cell proliferation in both EOL-1(F/P-positive human eosinophilic cell line) and imatinib-resistance (IR) cells. Altogether, our data demonstrate that Lyn is a vital downstream kinase activated by F/P converged with IL-5 signals in CEL cells. Lyn activate and expand IL-5RA intracellular signaling through FIP1L1-PDGFRA/JAK2/Lyn/Akt network complex, provoking eosinophils proliferation and exaggerated activation manifested as CEL.

## INTRODUCTION

Hypereosinophilic syndrome (HES) is defined as an unexplained persistent eosinophilia exceeding 1500/mm3 eosinophils per liter for more than 6 months in combination with symptoms and signs of organ damage resulting from eosinophil infiltration. It includes different types of hematologic diseases with male predominance and age distribution of 20–50 year-old. The FIP1L1-PDGFRA fusion gene(F/P) induces constitutive PDGFRA kinase activation, and exists in about 10–20% of HES/CEL [1]. F/P-positive HES/CEL patients present with exaggerated eosinophils proliferation and thus end-organs impairment due to eosinophil invasion and granulation toxicity [1, 2]. As a driver oncogene, F/P induces eosinophil abnormal differentiation and clonal proliferation *in vitro*. Nevertheless, simple expression of F/P into mice bone marrow cells results in the generation of a myeloproliferative disease without striking eosinophilia [3]. Introduction of interleukin-5 (IL-5) was necessary to recapitulate typical features of HES/CEL, including hyper eosinophilia and tissue infiltration of eosinophils when co-expressed with F/P fusion gene [4]. But the underlying mechanism was not yet fully understood.

IL-5 is a critical factor for eosinophil production and activation, but animal model showed that CD2-IL-5 Tg induced eosinophilia in blood, not in tissue [5]. Recently, some reported that amplification of IL-5 signaling by F/P triggers a CEL-like disease [6, 7]. Likewise, IL-5 can enhance blood eosinophil responsiveness to a second stimulus or event, resulting in a synergistic response, which was a radical mechanism of severe eosinophil-related inflammation. The process of cooperation between IL-5 and second stimulus did focus on the changes in intracellular signaling events [8]. Therefore, we hypothesized that preferential eosinophil development and overacting eosinophils, as a causal role of tissue eosinophil infiltration in F/P-positive CEL, maybe correlated with the synergistic effects of IL-5 with the F/P oncoprotein.

The IL-5 receptor A (IL-5RA) subunit is very important for IL-5 pathway and eosinophil development [9]. Lyn is physically associated with the IL-5RA chain upon receptor ligation, which appears critical for the subsequent activation of downstream signal pathway [10].

Autoinhibitory WW-domain of PDGFRA gene disrupted by the fusion of FIP1L1 translated the constructive activation kinase of PDFGRA into intracellular signal cascade [11]. The activated PDGFRA can bind and activate the signal molecules consisting of SH2-domain in cytoplasm [12]. Lyn is a member of Src family and characteristic of SH2 domain in addition to the kinase domain, and it stimulates PDGFR-induced malignant hematopoiesis [13]. Some reports about the downregulation of phosphorylated Lyn with EOL-1 (F/P-positive human eosinophilic cell line) cell growth inhibition in dasatinib treatment indicate that Lyn is the downstream of F/P oncoprotein [14]. But it was still unknown whether Lyn was involved in F/P-induced CEL.

First, we tested the idea if Lyn kinase was engaged in F/P signal-triggered CEL malignant phenotype, and whether Lyn activation in F/P-positive cell was strengthened by IL-5 cytokine. Then, we investigated whether Lyn can bind with Janus kinase 2 (JAK2) in F/P-spurring signal cascade [15]. Finally, we explored if Lyn kinase was in the F/P signal propagation, leading to the facilitation of IL-5RA intracellular signal pathway.

The results indicate that Lyn is a key downstream of F/P oncoprotein, which is responsible for F/P-stimulated eosinophils proliferation and apoptosis retardance. Moreover, the Lyn kinase serves as a critical component for imposing F/P-stirred eosinophil activation and major basic protein (MBP) release in response to IL-5 stimulation. Lyn can induce tyrosine phosphorylation of IL-5RA in F/P-expressing cells, and then facilitate the IL-5RA signal pathway to promote reinforced eosinophils function. The complex of F/P/JAK2/Lyn/Akt was identified in the F/P-expressing cells, and its disruption with combined inhibition of JAK2 and Lyn led to a dramatic depression of cell proliferation. These results also provide a potential alternative therapy for F/P-positive CEL.

## RESULTS

### Excessive phosphorylation of Lyn in F/P-positive CEL patients

**Figure 1:**
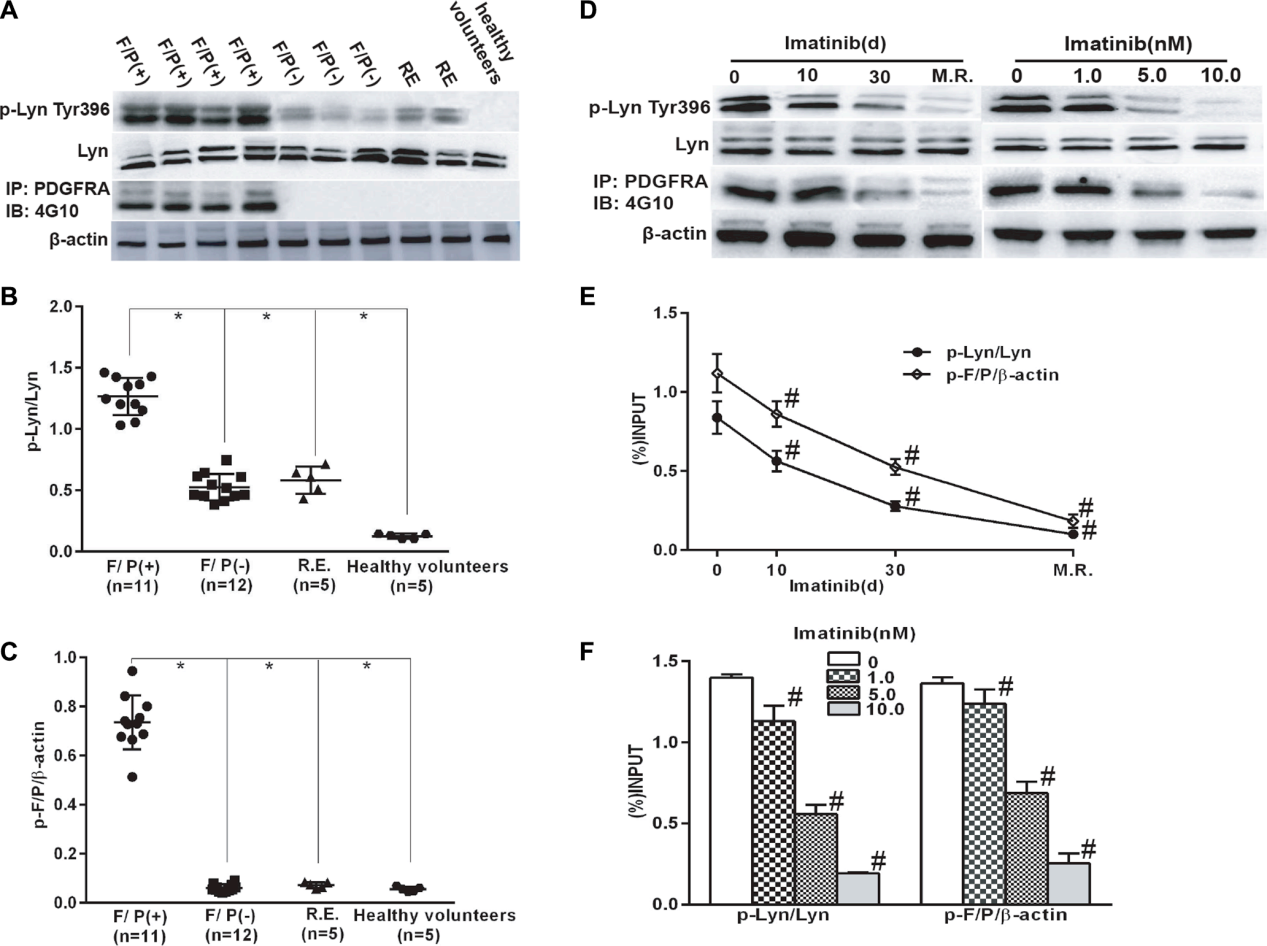
Lyn activation in F/P(+) CEL patients and was inhibited with the reduction in phosph-F/P by imatinib treatment Cell extracts were prepared from blood samples of the cases and subjected to Western blotting or immunoprecipitation (IP). (**A**, **D**) Reprsentative gell images. (**B**–**C**) Quantification of A. (**E**–**F**) Quantification of D. Data(mean ± SD) represent of the cases(F/P(+) CEL[*n* = 11], F/P(−) HES [*n* = 12], RE [*n* = 5], and healthy volunteers[*n* = 5]), or derived from three independent experiments.*P < 0.05, F/P(+) group compared to the other groups. F/P(+):F/P(+) CEL, F/P(−):F/P(−)HES,RE: reactive eosinophilia. ^#^*P* < 0.05, compared to day 0 or the untreated group.

We explored if Lyn was excessively activated in the F/P-induced CEL. The study included twenty-three HES cases. Polymorph nuclear leucocytes, as well as eosinophils were gathered for the immunoblotting [15]. The results displayed that the level of phospho-Lyn in F/P-positive [F/P(+)] CEL were higher than that in other eosinophilia cases without F/P gene (Figure 1).

### Lyn activation was inhibited with imatinib treatment in F/P(+) CEL patients and EOL-1 cells

Imatinib therapy is effective in F/P-positive cells and is the first choice for initial treatment in F/P(+) CEL patients. The patients with F/P gene were initially prescribed imatinib, and molecular remission was achieved in about 3–12 months after imatinib intervention. Proteins were extracted from the peripheral blood samples which were collected at four different time-points: day 0 (pre-therapy), day10 and day 30 of post-therapy, and the day of molecular remission (M.R.) [15]. At the same time, EOL-1 cells were treated with different doses of imatinib. The results demonstrated that phospho-Lyn level was gradually reduced in the F/P(+) CEL cases and EOL-1 cells with phospho-F/P inhibited by imatinib (Figure 1), which suggested Lyn kinase is the target of F/P oncoprotein.

### Lyn inhibition suppresses colony formation, induces apoptosis and prevents migration in response to IL-5 in F/P-positive CEL cells

**Figure 2:**
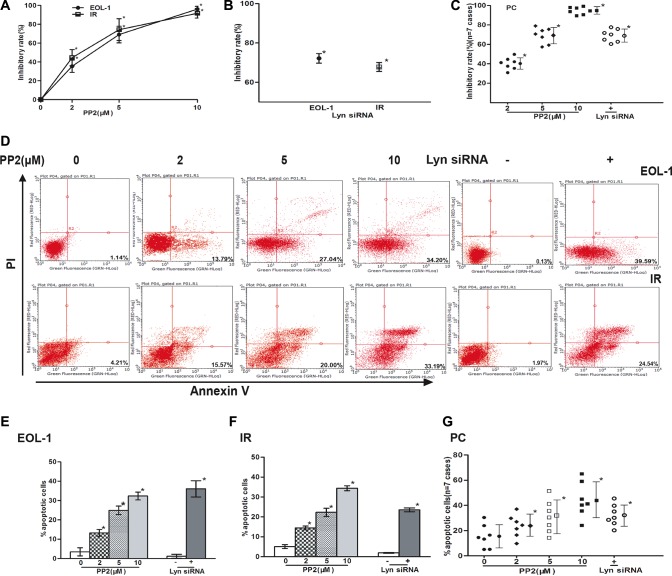
Lyn inhibition suppressed colony formation, induced apoptosis in EOL-1,PC and IR cells Cells were treated with various concentrations of PP2 or transfected with Lyn siRNA. Colony formation was assessed by soft agar colony-forming experiments, apoptosis assessed by flow cytometry using Annexin-V and PI. (**A**–**C**) Inhibitory rate of cells treated with various concentrations of PP2 or transfected with Lyn siRNA. (**D**) Representative flow cytometry patterns. (**E**–**G**) Percentages of apoptotic cells treated with various concentrations of PP2 or transfected with Lyn siRNA. Data (mean ± SD) representative results derived from three independent experiments [EOL-1 and IR] or the seven F/P-positive CEL patients.*P < 0.05,compared to untreated group or control siRNA.

To determine whether Lyn promotes cell proliferation, inhibits cell apoptosis and stimulates migration of F/P-positive CEL cells, we inhibited or knocked down Lyn with specific inhibitor PP2 or Lyn siRNA, and then assessed the clone-forming, cell apoptosis and migration. The results showed that colony formation was gradually inhibited with increasing dose of Lyn inhibitor, as well with Lyn knockdown in EOL-1 cells (Figure 2). The clone-forming of PC and IR cells were also dramatically inhibited after Lyn inhibition or knockdown (Figure 2). Lyn inhibition or knockdown in EOL-1 cells led to obvious cell apoptosis or even death. Similarly, PC or IR cells were also very sensitive to Lyn inhibition or knockdown (Figure 2 and Supplementary Figure S2). To explore whether Lyn stimulates migration of F/P-positive cells, IL-5 cytokine was used as chemoattractant in these cells. The cell migrations were suppressed when Lyn was inhibited by PP2 or knockdown with special Lyn siRNA (Supplementary Figure S3). Taken together, these observations suggest that proliferation, survival, and migration in F/P-positive cells maybe depend on cellular Lyn kinase activity, and Lyn molecule can be an alternative feasible treatment target for imatinib-resistant CEL. Notably, the level of phospho-Jak2 was not decreased by Lyn inhibition (Supplementary Figure S4), which indicated that JAK2 was not downstream of Lyn.

### IL-5 stimulation reinforced F/P-induced lyn activation

**Figure 3:**
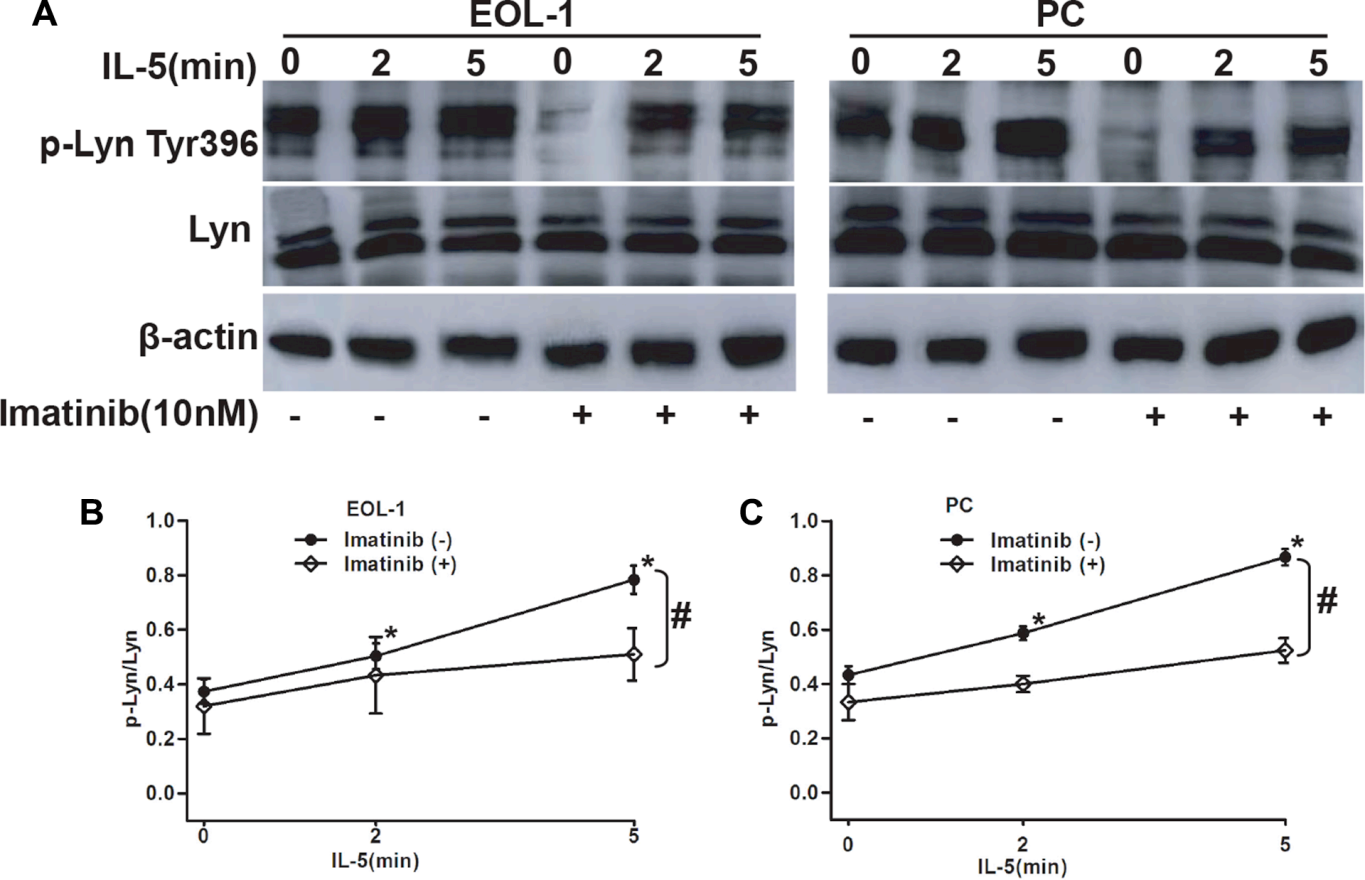
The synergistic role of F/P and IL-5 in inducing Lyn activation in EOL-1 and PC cells The EOL-1 or PC cells were preincubated with or without Imatinib for 4 h followed by treatment with 5ng/mL IL-5 for 0 to 5 min. Whole-cell lysates were prepared and subjected to Western blotting. (**A**) Representative gel images. (**B**–**C**) Quantification of A. Data (mean ± SD) representative results derived from three independent experiments. *P < 0.05, as compared to 0 minute. ^#^*P* < 0.05, compared to the differences between the Imatinib treatment and non-treatment for all the doses.

IL-5 cytokine is necessary for developing full CEL picture in the coordination with F/P oncoprotein. Lyn, as a critical mediator of IL-5 signaling pathway, was also proved to be activated by F/P oncoprotein in our previous findings. So we hypothesized that Lyn is the common target by F/P cooperated with IL-5 intracellular pathway in F/P-positive cells. To verify this hypothesis, we stimulated EOL-1 cells with IL-5 cytokines with or without 10 nM imatinib treatment. Our findings displayed that IL-5 stimulation dramatically increased phospho-Lyn level in F/P-expressing CEL cell, which can be obviously suppressed by imatinib, suggesting a collaborated activation of Lyn by F/P and IL-5 (Figure 3).

### Lyn inhibitor or knockdown suppresses IL-5-stimulated MBP release and the activation of PI3K/Akt pathway

**Figure 4:**
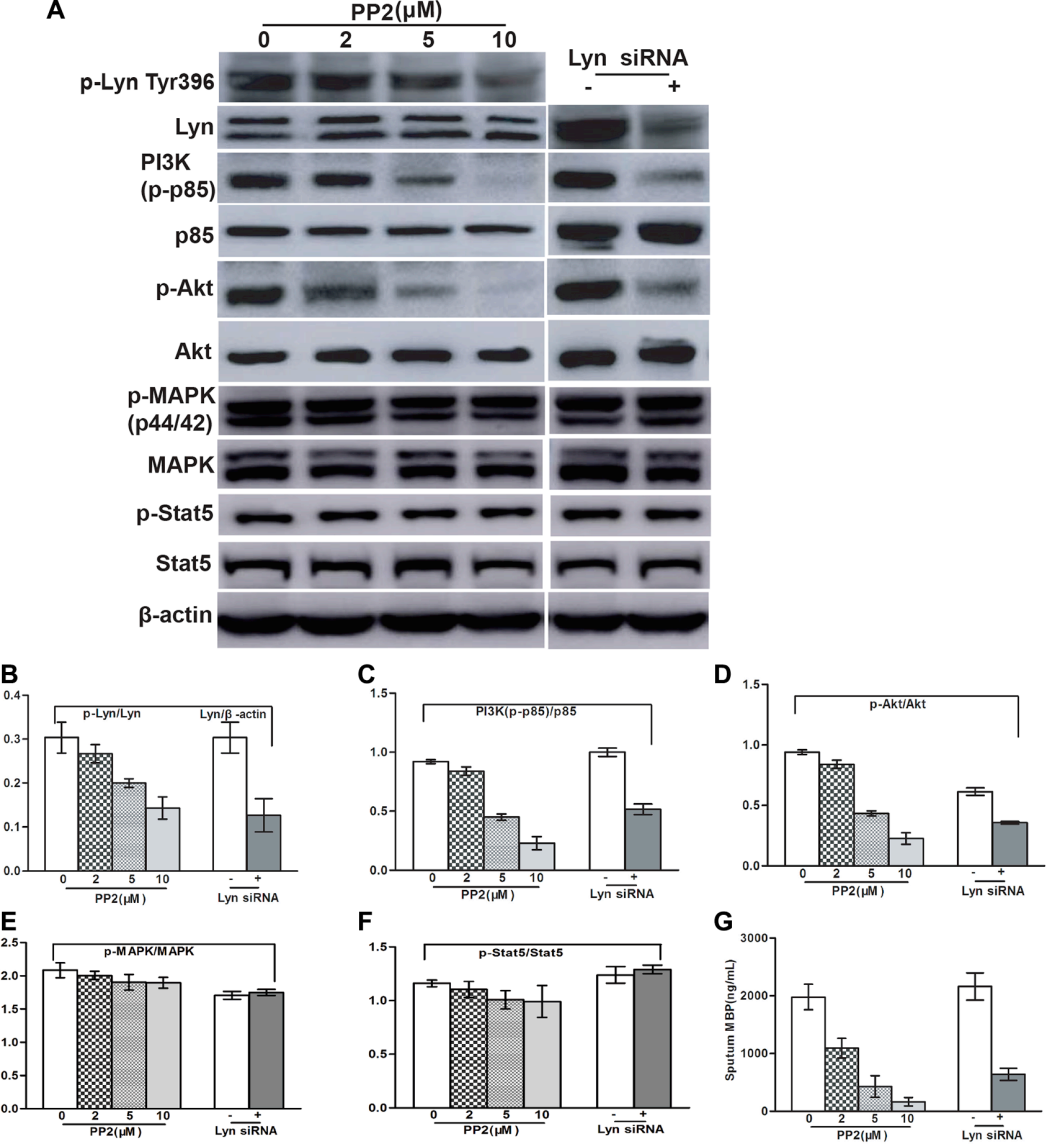
Lyn inhibition blocked IL-5-induced MBP release and the downregulation of PI3K/Akt in EOL-1 cells The EOL-1 cells were treated with various concentration of PP2 or transfected with Lyn siRNA. (**A**) Representative gel images. (**B**–**F**) Quantification of A. (**G**) MB *P *values were determined using a double antibody competitive radioimmunoassay.

MBP is the major cytotoxic granule in eosinophil, which takes key responsibility for eosinophil-associated organ damage depending on the activation of PI3K/Akt molecules [16]. Lyn was shown to be the focus downstream of cytokine IL-5 and F/P oncoprotein, and then we assessed whether Lyn participate the cooperation effect of IL-5 and F/P inducing eosinophil activation and PI3K/Akt activation. The results showed that Lyn inhibition strikingly suppressed IL-5-stimulated MBP release and PI3K/Akt activation in F/P-positive CEL cells (Figure 4). But there was no difference in the signal transducer and activator of transcription 5 (Stat5) or extracellular-regulated kinase (ERK) activation with or without IL-5-stimulation in F/P(+) EOL-1 cells (Figure 4). PI3K inhibitor GDC 0941, but not MEK inhibitor [PD 325901], reduced the MBP release of EOL-1 and IR cells (Supplementary Figure S5). These findings indicate that excessive Lyn activation endows the invasive power of F/P-positive eosinophils by activating PI3K/Akt pathway.

### Lyn binds to the cytoplasmic region of IL-5RA and induces its tyrosine phosphorylation in F/P-expressing cells

**Figure 5:**
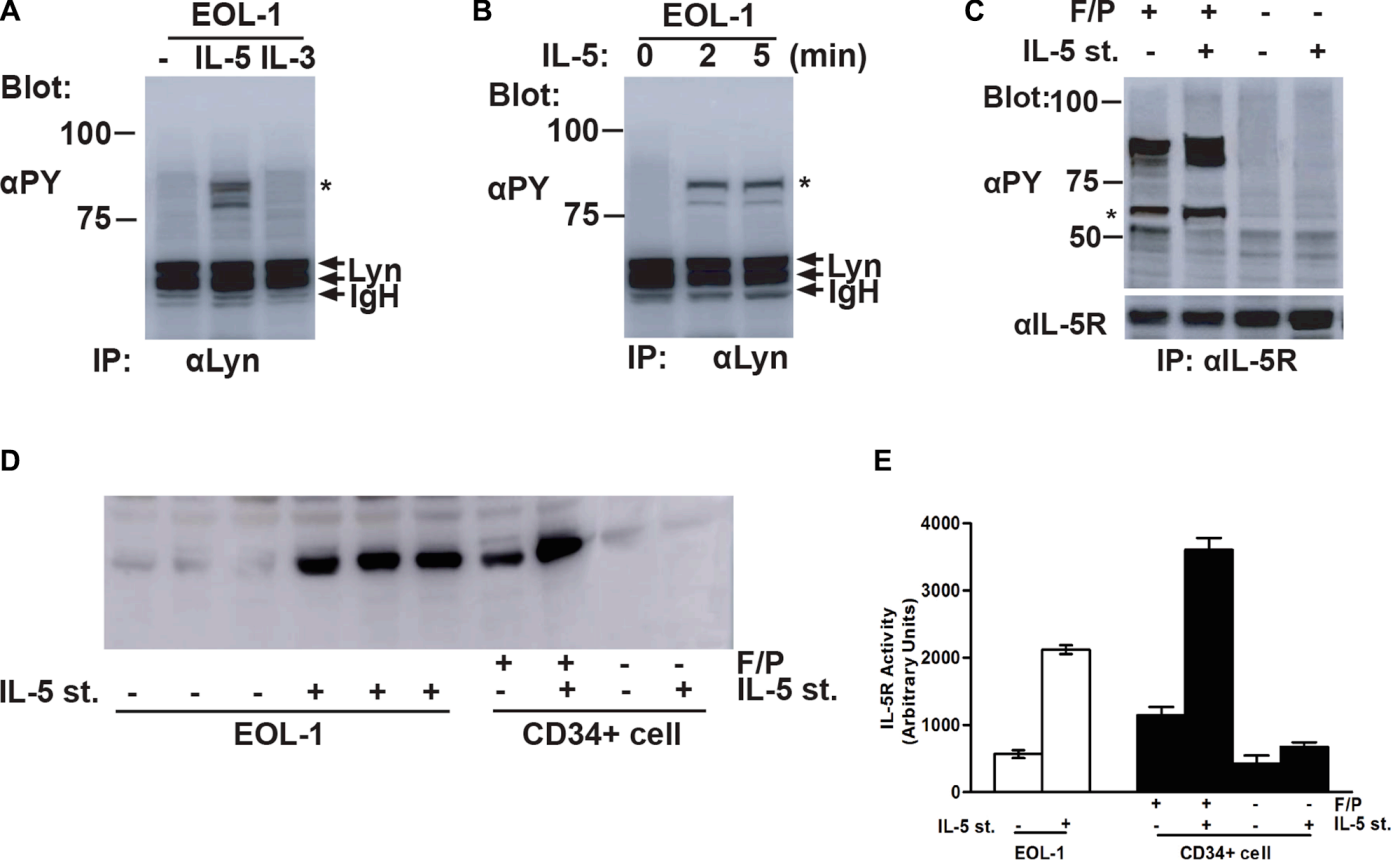
Lyn induced tyrosine phosphorylation of IL-5RA and associated with the phospho-IL-5RA in F/P-expressing cells (**A**) EOL-1 cells were starved overnight and left unstimulated (−) or stimulated with 5 ng/mL of IL-5 or 25 ng/mL of IL-3(IL-3) for 5 minutes at 37°C before solubilization. Cell lysates were immunoprecipitated with anti-Lyn. Immunoprecipitates were resolved by SDS-PAGE and subjuected to immunoblotting with an anti-phosphotyrosine monoclonal antibody, 4G10(αPY). (**B**) EOL-1 cells was starved overnightand stimulated with 5 ng/mL of IL-5 for the indicated times. Cells were then lysed and analyzed as described above. (**C**) In CD34+ cells, the IL-5R was transiently coexpressed with FIP1L1-PDGFRA as indicated. Transfected cells were either stimulated with 5 ng/mL of IL-5 for 5 minutes or left unstimulated, as indicated(IL-5 st. + or −, respectively).Cells were lysed and immunoprecipitated with anti-IL-5Rα. Immunoprecipitates were analyzed by anti-phosphotyrosine(αPY) Immunoblotting followed by reprobing with anti- IL-5R, as indicated. A coimmunoprecipitated phosphotyrosyl protein that corresponds in size to Lyn is indicated with an asterisk. (**D**) Immunopurified IL-5R was subjected to vitro kinase assays. (**E**) Quantification of D.

Although animal model indicated that the crucial synergistic effect of IL-5 on the whole F/P-triggered CEL characteristics, serum IL-5 level was usually normal in F/P-positive cases [17]. The consensus of the forgoing function of IL-5 is attributable to the signaling via the IL-5RA. F/P signal pathway sensitize IL-5R-positive myeloid progenitor and precursors to IL-5 stimuli, may be by a presumed up-regulation role on IL-5RA of F/P-expressing cells, imposing eosinophil production and activation [4]. Therefore, it is plausible that activated intercellular Lyn kinase can bind with IL-5RA bringing about and enhancing the activation of IL-5RA signaling pathway. The other evidence supporting our hypothesis is that Lyn is correlated with different transmembrane receptors, including IL-5R, in diverse cell categories [10, 18]. To address this concern, we stimulated F/P(+) EOL-1 cells with IL-5. A phosphorylated protein corresponding in size to IL-5RA was shown to be co-immunoprecipitated with Lyn when IL-5 cytokine was added (Figure 5A). We also detected that phospho-IL-5RA combination to Lyn protein was increasing upon the IL-5 stimulation time in EOL-1 cells (Figure 5B). And then, we ask if Lyn can stimulate the activation of IL-5R, IL-5R was co-expressed with F/P into CD34 (+) cells. IL-5 could not stimulate observable phospho-IL-5RA level when the cells were only introduced with IL-5RA. But F/P and IL-5RA co-expression promoted a conspicuous activation of IL-5R despite the lack of IL-5 induction (Figure 5C). The level of tyrosine phosphorylation of the IL-5R was significantly enhanced by IL-5 stimulation. *In vitro* kinase assay was applied for the detection of IL-5R kinase. Compared with unstimulated or untransfected cells, the IL-5R tyrosine kinase was significantly enhanced by approximately 273.4% or 434.3% respectively (Figure 5D–5E). The phosphorylated protein equivalent to the molecule weight of Lyn was co-immunoprecipitated with IL-5R in the cell co-expressing F/P and Lyn (Figure 5C). Interestingly, the level of phospho-Lyn protein was reinforced by IL-5 stimulation (Figure 5C).

### Lyn is associated with FIP1L1-PDGFRA/JAK2 signaling complex in F/P-expressing cells

**Figure 6:**
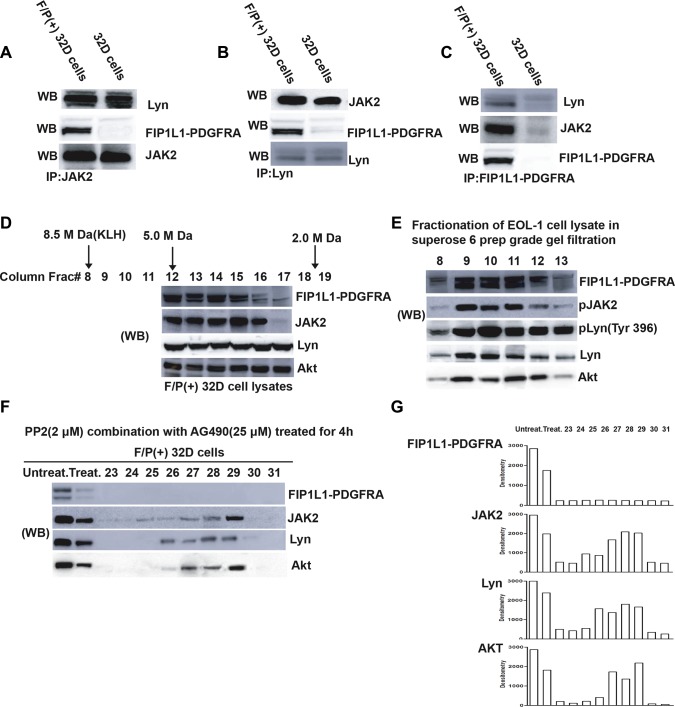
Detection of a large molecular weight signaling network complex comprised FIP1L1-PDGFRA, Jak2, Lyn and other proteins (e.g. Akt) in F/P-expressing cells (**A**–**C**) Lyn and JAK2 proteins are parts of FIP1L1-PDGFRA network. F/P(+) 32D cells were immunoprecipitated with several antibodies, and the immunoprecipitates were detected by Western blotting. (**D**) Proteins from the detergent extracted lysate of F/P/(+) 32D cells were eluted from the column by a detergent-containing buffer.From each eluant, a 25 μl aliquot was taken and analyzed by western blotting, and the membrane was probed with different antibodies as indicated. (**E**) Fractionation of proteins from the EOL-1 cell lysates on a gel filtration column, as described in Figure 6D. Detection of signaling molecules in the colum eluant was performed by western blotting. (**F**) Treatment of F/P(+) 32D cells with 25 μM AG490 and 2 μM PP2 for 4 hours disrupted the FIP1L1-PDGFRA/JAK2/Lyn network structure. The procedure for analysis was the same as used for (D). (**G**) Densitometric analysis of the Wb bands of the immunoblots of (F).

Our previous study has revealed that JAK2 was involved in F/P-stimulated eosinophil proliferation and migration [15]. To identify the F/P-associated proteins, detergent lysates of the mouse 32D cells and F/P(+) 32D cells were analyzed by immuno-precipitation with either F/P antibody-protein A conjugate (for F/P-associated proteins) or by the addition of other specific antibodies(e.g., JAK2, or Lyn). These same antibodies were used to identify F/P network-associated proteins by Western blotting of the immuno-precipitates. Immunoprecipitation and Western blotting of the detergent-soluble lysates from either 32D cells maintained in IL-3 or F/P(+) 32D cells with the anti-JAK2 antibody detected Lyn and F/P in the F/P(+) 32D lysate. If the same lysates were used for immunoprecipitation with anti-Lyn and probed with the respective antibodies, F/P [only in the F/P(+) 32D lysate] and JAK2 were detected. Immunoprecipitation with anti-F/P of these lysates followed by Western blotting with the appropriated antibodies identified JAK2, and Lyn, the downstream effectors of F/P kinase, only in the F/P(+) 32D lysate (Figure 6A–6C). Thus, F/P protein network constitutes a signaling complex, which contains JAK2, and Lyn. Other proteins likely to be in the F/P/JAK2/Lyn network should be further studied.

### Identification of F/P/JAK2/Lyn/Akt complex in F/P-expressing cells and combined inhibition of the Lyn and JAK2 dramatically blocks cell proliferation via complex disruption

Akt hyperactivation was detected from F/P-positive cells [19, 20]. Therefore, we hypothesized the possibility of F/P/JAK2/Lyn/Akt network complex in the F/P-induced CEL. To address this concern, Gel filtration column chromatography and proteomics were performed [18]. Several molecules, such as Akt, pLyn, Lyn and JAK2 were observed in the same eluent (Figure 6D). Other downstream proteins, including pERK, pSTAT5, STAT5, should be detected in the further study. The complex was also detected in EOL-1 cell (Figure 6E). When EOL-1 or IR cells were treated with low dose of AG490 (25 μM) and PP2 (2 μM), the cellular proliferation was more significantly blocked, compared with either AG490(25 μM ) or PP2(2 μM) by themselves(data not shown). Then we ask if F/P/JAK2/Lyn/Akt network complex can be disrupted by the combined inhibition of JAK2 and Lyn, F/P(+) 32D cells were incubated with 25 μM AG490 and 2 μM PP2 for 4 hours followed by Gel filtration column experiments. This broken F/P/JAK2/Lyn/Akt complex was indicated in Figure 6F which showed that F/P oncoprotein and the rest of the complex components were significantly decreased in quantity. These results further provide evidences that F/P-incited intracellular signal, center on its downstream of JAK2/Lyn, which then activating and recruiting the other downstream of F/P such as Akt, and finally leading to the CEL malignant traits. Moreover, disruption of the F/P/JAK2/Lyn/Akt complex with dual inhibitors may become a realistic therapeutic options for the F/P(+) CEL.

## DISCUSSION

The F/P fusion gene which leads to the constitutive activation of PDGFRA drives the occurrence of CEL [1]. But the whole CEL characteristics with eosinophilia-associated end-organ infiltration needs oncoprotein F/P collaborated with additional IL-5 stimulation [4, 21]. The mechanisms underlying the communication between F/P-activated molecules and IL-5-stimulated intercellular signal molecule were still unknown. Here, our findings first identified that Lyn is hyperactivated in F/P-positive CEL and is an important kinase in F/P-induced cell survival. Lyn inhibition can repress cell migration and MBP release. We further delineated that Lyn can induce IL-5RA tyrosine phosphorylation and physically associate with IL-5RA in F/P-expressing cells. Finally, the complex of F/P/JAK2/Lyn/Akt was identified in F/P-expressing cells, which can be disrupted by double inhibition of JAK2 and Lyn, leading to significant suppression of proliferation in these cells. These results provide the feasibility of targeting both JAK2 and Lyn in treatment of F/P-positive CEL with imatinib-intolerance or IR, and also offer a new insight into the mechanism of F/P-driven CEL.

Lyn kinase has been implied as important actor for eosinophil proliferation and function in response to IL-5-stimulation [10, 22, 23], we evaluated whether Lyn kinase was activated by F/P oncoprotein in CEL. As expected, the results displayed that all the F/P-positive CEL patients expressed higher level of phospho-Lyn than other F/P-negative subjects. The phosphorylated Lyn (p-Lyn) was inhibited with the suppression of phosphorylated F/P (p-F/P) by imatinib in a dose- and time-depended manner. This result places Lyn in the downstream of F/P oncoprotein. F/P-transformed cells undergo cytokine-independent proliferation and delayed apoptosis, being ascribed to its activating a common set of downstream molecules including ERK1/2, STAT5 and PI3K [1, 24–26]. However, these intercellular molecules cannot take full responsibility for the oncogenic role of F/P in CEL [1, 25]. As it can be directly bound and activated by PDGFR, Lyn has been proposed as a key actor in leukemogenesis through the recruitment of divergent intracellular signaling pathways [27–29]. Our results showed that Lyn inhibitor can dramatically reverse the function of F/P fusion gene promoting proliferation and anti-apoptosis of EOL-1 cells, which indicated Lyn as an important player in the F/P-positive CEL.

Not BCR/ABL oncogene, but F/P oncogene is the co-stimulator for IL-5 to produce CEL [4]. Synergistic activation of cytokine-stimulated signaling pathways in the presence of other factors, has been observed in various cell types [30]. In eosinophils, priming with IL-5 family members is known to increase cellular responsiveness to chemoattractant and render a hypersensitive phenotype [8, 31]. Lyn is not only important for IL-5-induced signaling and cellular responses, but also as a key ingredient in the crosstalk between IL-5 and chemotactic factors [22]. If both cytokine IL-5 and F/P oncogene were introduced into the CD34+ cells, the overwhelming characteristics of F/P-positive CEL will be fully presented with end-organ impairment from eosinophil infiltration and activation [4]. The capacity of IL-5 to prime blood eosinophils hypersensitive to second stimulus has been associated with changes in intracellular signaling events. Several lines of evidences indicated that Lyn as one of the key players in the hyperactive phenotype of IL-5 primed blood eosinophils [22, 32]. Our study showed that IL-5 can induce distinct augment of p-Lyn in F/P-expressing eosinophil cells, which was partially prevented by pre-incubation with specific F/P inhibitor, imatinib. These results suggested the coordinated activation on Lyn signaling pathway from F/P oncoprotein with IL-5 cytokine. Additionally, there were no significant differences neither in the levels of p-F/P nor IL-5RA activation after IL-5 stimulation, which showed no direct evidence for trans-activation between IL-5RA and F/P molecule themselves [Data no shown]. Neither was there any hyperactivation of STAT5 or ERK by F/P cooperation with IL-5 [Data no shown].

Clinically, the CEL patients carrying F/P oncogene have a serious and extensive end-organ impairment resulting from the eosinophil infiltration and subsequent fibrosis [2]. Mostly, the eosinophils-related heart damage become the primary cause of morbidity and mortality in F/P-positive CEL [17]. MBP, one of the most potent eosinophilic granule proteins, is the major causes leading to severe target-organ damage [33].

Our data showed that MBP release was reduced with Lyn inhibition in EOL-1 cells. Lyn oncoprotein can promote induction of myeloproliferative neoplasm with myelofibrosis [34, 35]. Although the molecular mechanism was not yet clear, the results indicated that Lyn inhibition may become another possible strategy on blocking F/P-induced eosinophil activation and thus eosinophil-related end-organ damage.

PI3K/Akt pathway was correlated with direct degranulation and superoxide production in the effect of IL-5 and F/P on blood eosinophils [20, 36], we were inspired to probe whether PI3K/Akt kinase can be activated by Lyn, conducting F/P-positive eosinophil invasion. As expected, our data showed that the phosphorylation level of PI3K and Akt proteins was strikingly decreased with Lyn inhibition in EOL-1 cells, which shed light on the F/P-induced end-organ eosinophil infiltration being through Lyn/PI3K/Akt pathway. STATs or ERK molecules are another important downstream effectors of IL-5 signal pathway during eosinophil development. However, our findings showed no enhanced activation of Stats or ERKs in F/P-positive CEL cells upon IL-5 stimulation. Thus, it is presumable that the synergism of F/P and IL-5 promotes aggressive eosinophil infiltration and activation by converging into intracellular Lyn/PI3K/Akt pathway differing from those in normal eosinophil biological function.

Clinically, some F/P-positive CEL patients were sensitive to anti-IL-5 treatment albeit the serum IL-5 concentrations of them were within the normal range. It supposed that happen via direct or an assumed activation role on IL-5R in F/P-expressing cells [4, 6]. Therefore, we investigated whether Lyn can be associated with IL-5RA and induce a presumed IL-5R activation of the F/P-expressing cells. The results showed that Lyn can physically associate with the IL-5RA in EOL-1 cells. Further study displayed that IL-5 cannot stimulate discernible IL-5RA activation when only IL-5RA was introduced into the CD34+ cells. However, the co-expression of FIP1L1-PDGFRA with IL-5R induced a prominent tyrosine phosphorylation of IL-5RA and Lyn, which was stronger upon IL-5 stimulation.

Membrane targeting is known to be critical for controlling Lyn activity [37]. F/P-triggered signal cascade may lead to the recruitment of Lyn into membrane domains, and increase the basal activity of Lyn, which binds with specific IL-5R subunit, and thereby activate and amplify IL-5RA-related pathway.

The processes by which Lyn contributes to the crosstalk between IL-5 and F/P- signal are not fully understood. JAK2 kinase is recognized as the primary initiator of IL-5R signaling, whereas Lyn has been implicated as a key ancillary kinase [38]. JAK2 was the downstream of F/P oncoprotein and enforced JAK2 activation upon IL-5 stimulation was also verified in F/P-related pathway [15]. It motivated our interest to explore whether Lyn could bind with JAK2 in F/P-positive EOL-1 cells. The other evidence supporting our study is that the fact about Lyn SH2 domain can direct bind to tyrosine-phosphorylated JAK2 [39]. JAK2 inhibition can inactivate Lyn kinase in BCR/ABL-induced Chronic Myeloid Leukemia (CML) [40, 41]. As expected, our study showed that Lyn protein can be detected in the anti-JAK2 immunoprecipitation complexes, besides it occurred in the anti-F/P-immunoprecipitation complexes in F/P-transfected 32D cells. These results revealed that Lyn is associated with JAK2 molecule in F/P-activated signal cascade.

It was well-established that oncoproteins like BCR-ABL, as well as PDGFR is combined to Src family and its downstream molecules [42, 43]. Therefore, we detected whether the signaling network including JAK2, Lyn, Akt and F/P exists in the F/P-expressing cells. The gel filtration experiments in Figure 6 support this hypothesis of F/P/JAK2/Lyn/Akt complex in F/P(+) 32D cells and EOL-1 cells.

F/P-positive cells are sensitive to imatinib *in vitro* and vivo. But imatinib resistance will eventually occur after long-time treatment for F/P-positive CEL, and imatinib has a possible cardio toxicity [14]. Moreover, the limited clinical activity of other TKIs highlights the need to develop new alternative strategy for blocking the growth of neoplastic eosinophils [44].

**Figure 7:**
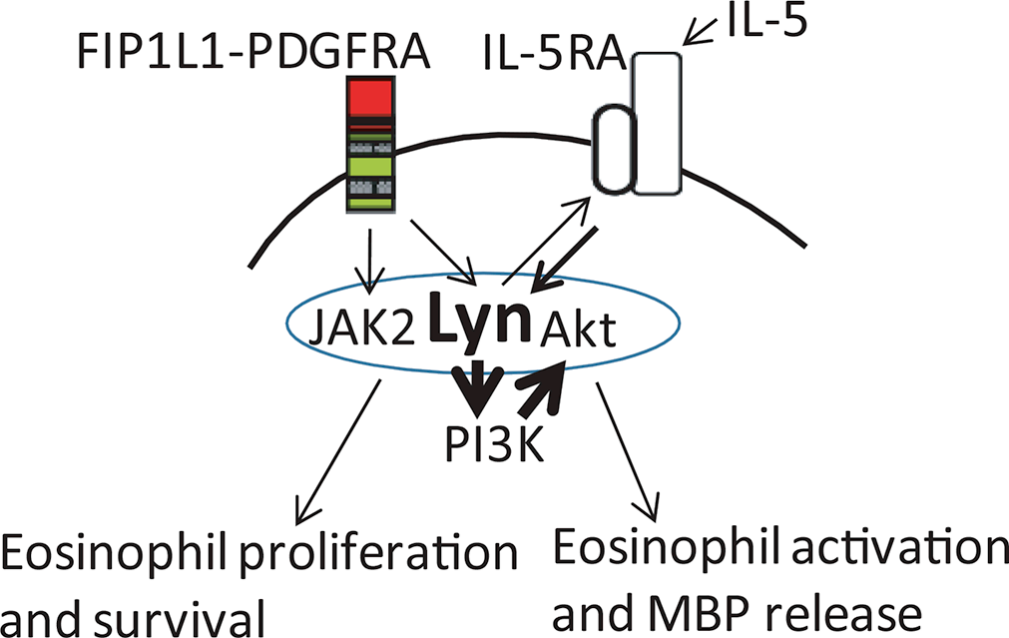
FIP1L1/PDGFRA in conjunction with IL-5 promote eosinophil proliferation and excessive activation Systemic or local (paracrine or autocrine) IL-5 effects and indirectly through a putative up-regulation effect on IL-5RA signal pathway facilitation its intracellular signaling in F/P(+) cells. Lyn kinase was activated by F/P, and then associated with and phosphorylated IL-5RA, ease and amplify IL-5RA signal pathway.

Recent reports show that dasatinib, a double inhibitor for Lyn and PDGFRA kinase, have the same or even more effectiveness than imatinib in F/P-positive CEL [14, 45–47]. Therefore, the F/P-positive cells were treated with the combination of low-dose JAK2 inhibitor and Lyn inhibitor, the cell proliferation was expectantly inhibited. The network complex of F/P/JAK2/Lyn/Akt was remarkably interrupted. Although the underlying mechanism is still unclear, we emphasize that the demolition of F/P/JAK2/Lyn/PI3K complex do result from the inhibition of Lyn kinase and the rapid reduction of JAK2 protein levels. We propose a schematic diagram depicting Lyn activation of F/P-induced pathways to stimulate eosinophils proliferation and activation (Figure 7).

In summary, we reveals that Lyn was activated by F/P oncoprotein. Moreover, the results feature Lyn as a significant participator for the pathogenesis of F/P-positive CEL. Lyn is also critical for the enhanced release of MBP in F/P-induced eosinophils upon IL-5 stimulation. More importantly, this study underscores that Lyn maybe stimulates the hyperactive phenotype of F/P-positive cells in response to IL-5 stimulation, by associating with IL-5RA and subsequently priming its signaling pathway, which in turn contribute to the increased proliferation and infiltration capacity of F/P-positive eosinophils. The identification of F/P/JAK2/Lyn/Akt complex emboldens us to hypothesize that Lyn may act as a signaling node, converging the F/P and IL-5 signals, which subsequently initiates and spreads multiple output signals such as MEK/ERK and PI3K/Akt pathway [25]. Collectively, this study suggests that Lyn may be a potential therapeutic target for F/P-positive CEL patients with imatinib intolerance or resistance. These study disclose the molecular mechanisms of the crosstalk between oncogene and cytokine, which represents a model to study cellular integration of biochemical signals in cancer, being responsible for crucial aspects of cancer biology.

## MATERIALS AND METHODS

The present study protocol was approved by the ethical committee at Xiangya Hospital of Central South University, Changsha, China.

### Patient samples

A total of 28 patients, including 23 cases of HES, five of reactive eosinophilia (R.E., two of which were eosinophilic gastroenteritis and three were ancylostomiasis) and five healthy volunteers, were included in this study. Karyotype analysis was normal. No abnormal chromosomes, including those of PDGFRB, FGFR1 and JAK2, were detected in any of the cases. The 23 HES patients met all the criteria for the diagnosis of HES, as proposed by Chusid [48]. Nested RT-PCR and fluorescence *in situ* hybridization (FISH) analyses were performed on all samples, and the F/P fusion gene was detected in the 11 HES/CEL patients, but not in the other 12 HES patients or other subjects. 10 of the 11 F/P(+) CEL cases had organ involvement (three of which had one impaired organ and seven had at least two impaired organs). The concentrations of serum IgE and IL-5 were normal in all 11 F/P(+) CEL patients.

### Cell culture

EOL-1 cells carried the WT F/P fusion oncogene (Braunschweig, Germany) [49]. Ba/F3 cells expressing T674I F/P resistant to imatinib (IR) have been described previously [1]. After obtaining blood from the above-mentioned patients, polymorphonuclear leucocytes were separated by standard laboratory procedures [50]. Eosinophils were then separated by depletion of neutrophils with anti-CD16-coated magnetic microbeads using the magnetic cell separation system (MACS;Miltenyi Biotec GmbH, Bergisch- Gladbach, Germany). All these cell lines and primary cells were maintained in RPMI-1640 medium supplemented with 10% fetal bovine serum (FBS) (Hyclone, USA) at 37°C in a humidified atmosphere of 5% CO2. IL-3-dependent 32D cells were maintained in RPMI 1640 medium supplemented with 10% fetal calf serum(FCS) and 10% WEHI-3 conditioned medium as a source of IL-3 [51]. MACS immunomagnetic cell separation (Miltenyi Biotech, Auburn, CA) using a hapten-conjugated antibody against CD34, which was coupled to beads, was used to isolate CD34+ cells. CD34+ cells were cultured in Iscove’s modified Dulbecco’s medium (Life Technologies, Paisley, United Kingdom) supplemented with 8% FCS, 50 Amol/L of h-mercaptoethanol, 10 units/mL of penicillin, 10 Ag/mL of streptomycin, and 2 mmol/L of glutamine at a density of 0.3 × 10^6^ cells/mL.

### Stimulation of cells with IL-5

EOL-1 or PC were preincubated with or without imatinib (10 nM) for 4 h and stimulated with IL-5 (5 ng/mL at 37°C for 0 to 5 min). The phosphorylation level of Lyn was detected by Western blotting at the time points of 0, 2 and 5 min after IL-5 stimulation.

### Construction of lentiviral vectors

The shRNA sequence targeting Lyn and scrambled control shRNA sequence were designed and synthesized (GenScript, USA). The sequence information is listed below in detail: Lyn siRNA1: 5′-AGAUUGGAGAAGGCUUGUAUU-3′; 5′-AAUACAAGCCUUCUCCAAUCU-3′; Lyn siRNA2: 5′-CCAUCACUGGUUGCACUUAUU-3′; 5′-AUAAGUGCAACCAGUGAUGG-3′; Lyn siRNA3: 5′-AGUAUUCUGUACUCUUAGAUU-3′; 5′-AUCUAAGAGUACAGAAUACU-3′. Scrambled control shRNA sequence: 5′-UUGUACCUAAUUUCGUCCCAC-3′; 5′-UGGGACGAAAUUAGGUACAA-3′. Lentiviral DNA constructs were used co expressing enhanced green fluorescent protein (eGFP) and either FIP1L1-PDGFRA, fusing the NH2-terminal 233 amino acids of FIP1L1 to the COOH-terminal 523 amino acids of PDGFRA [11], or/and IL-5RA [52]. Lentiviral vectors were packaged in T293 cells using reagent of the lipofectamine and plus^TM^, according to the manufacturers′ instructions (invitrogen, USA).

### Infection of cells with the lentiviral virus

Cells were cultured in the medium as above described. An appropriated amount of virus was diluted into the corresponding culture medium (containing 8 μg/ml of polybrene) . Different types of recombinant viral supernatant and control viral supernatant were added separately. Then, the old medium was exchanged for the medium containing the virus, and the cells were incubated overnight. The following day, the medium containing the virus was removed and replaced with fresh culture medium. Transduced cells were selected using puromycin or blasticidin. Western blotting were executed for detecting the knockdown efficacy of Lyn protein for different Lyn shRNAs in EOL-1 cells. Lyn siRNA1 was selected for the following experiments because of > 95% inhibitory rate for Lyn protein (Supplementary Figure S1). Transduction efficacy of F/P or/and IL-5RA into the cells was proved > 90% (Supplementary Figure S2).

### Colony formation assay

EOL-1, IR or PC cells were treated with different doses of PP2 or Lyn siRNA. After 72 hr, cells were seeded in triplicate into 35-mm dishes containing agarose and RPMI 1640. The plates were incubated at 37°C, in 5% CO2, for 2 weeks. Colonies (cell numbers ≥ 50 = colony) were counted and photographed. The inhibition rate was calculated as follows: Colony inhibition rate (%) = (1 -Average colony number in treated group/Average colony number in blank control) ×100%.

### Apoptosis assay

Apoptosis was measured by Annexin-V-FLUOS and propidium iodide (PI) double staining, according to the manufacturers′ instructions (BD Biosciences Pharmingen, USA) and analyzed with a FACScalibur flow cytometer and CellQuestPro software (BD Biosciences). Morphological identification of apoptotic cells was refer to the method of Nicolatti et al. [53]. Briefly, EOL-1, IR or PC cells were cultured and treated with different concentrations of Lyn inhibitor (PP2) or knockdown with Lyn siRNA as described above. After the end of incubation, cell were collected, washed, and stained by acridine orange/ethidium bromide (AO/EB) or incubated with Annexin-V and then propidium iodide for flow cytometric analysis.

### Eosinophil migration assay

Migration properties of EOL-1 and IR cells were analyzed in a 48-well microchamber (Neuroprobe, USA), in which the lower wells were filled with 28 μL of buffer alone or buffer containing 5 ng/mL IL-5. A fibronectin-coated polyvinylpyrrolidone-free filter (Neuroprobe) with 5 μm pores was placed over the lower wells and 50 μL of EOL-1 and IR cells at 4 × 10^6^ cells/mL was added to the upper wells. The chamber was incubated for 2 h at 37°C in a CO2 incubator. After the polycarbonate filter was removed and cells adhering to the upper surface were wiped off using a filter wiper. The filter was dried, fixed, and stained with Diff-Quick reagent (Baxter Diagnostics, USA). The cells in two randomly selected fields per well were counted using the Axiovert 25 microscope (Carl Zeiss, Germany). Each experiment included six replicate measurements. The chemotactic index (CI) was calculated as the number of cells that migrated in experimental wells compared to those in control wells (was set at 100%).

### *In vitro* kinase assays

EOL-1 cells were starved overnight and left unstimulated (−) or stimulated with 5 ng/mL of IL-5 for 5 minutes at 37°C before solubilization. For the IL-5R kinase assay, Cell lysates were immunoprecipitated with anti-IL-5R antibodies. After extensive washing, IL-5R immunoprecipitates were incubated with [γ −32P]ATP (15 Ci) for 30 min at room temperature in reaction buffer (20 mM HEPES [pH 7.6], 5 mM MnCl2, 100 mM NaCl, 0.5 mM dithiothreitol, 1 mM Na3VO4, 5 mM MgCl2, 20 M cold ATP, 10 g of aprotinin/mL, 10g of leupeptin/mL). The reaction was stopped by adding 10 mM EDTA. The reaction mixture was resolved by SDS-PAGE and subsequently subjected to autoradiography. In CD34+ cells, the IL-5R was transiently co-expressed with FIP1L1-PDGFRA as indicated. Transfected cells were either stimulated with 5 ng/mL of IL-5 for 5 minutes or left unstimulated, as indicated (IL-5 st. + or −, respectively).Cells were lysed and immunoprecipitated with anti-IL-5Rα. Immunoprecipitates were analyzed by anti-phosphotyrosine (αPY) Immunoblotting followed by reprobing with anti-IL-5R, as indicated. A coimmunoprecipitated phosphotyrosyl protein that corresponds in size to Lyn is indicated with an asterisk. IL-5R was immunoprecipitated with anti-IL-5R and subjected to an vitro kinase assay as Duan et al. described [54].

### Immunoprecipitation and immunoblotting

For immunoprecipitation, the cell lysates were incubated on ice with anti-Lyn, anti-PDGFRA anti-JAK2 or anti-IL-5RA antibody (1:100-1:1000 dilutions; Santa Cruz Biotechnology, USA) for 2 h. The immune complexes were collected following incubating with protein A-agarose (Roche, USA) at 4°C for 1 h. The beads were then washed three times with washing buffer and boiled for 5 min in SDS-PAGE sample buffer. The solubilized proteins were separated by SDS-PAGE, transferred to a nitrocellulose membrane (Amersham Biosciences, Sweden), and detected by immunoblotting against antiphosphotyrosine monoclonal antibody, 4G10 (αPY) or Western blotting was performed as described previously [15]. Blots were probed with the primary antibodies against phospho-Lyn (Y396) (p-Lyn), Lyn, phospho-p85a (PI3K)/(Tyr467)(p-p85a), p85a, phospho-Akt1 (Thr308/Ser473)(p-Akt1) and Akt1 (Santa Cruz Biotechnology, USA), phospho-Stat5(Tyr 694), and Stat5 (Invitrogen, USA), phospho-MAPK p44/42(Thr 202/Tyr 204), MAPK, phospho-JAK2 (Tyr1007/1008)(p-JAK2), JAK2 and β-actin (Cell Signaling Technology, USA) followed by incubation with the secondary antibodies were used peroxidase-conjugated goat antimouse IgG or goat anti-rabbit IgG (Jackson ImmunoResearch Inc., USA) and enhanced chemiluminescent substrate. Densitometry analysis was performed on exposed films using Quantity One v4.62 software (Bio-Rad, USA).

### Gel filtration column chromatography

The protein separation column selected for this purpose was 50 cm length × 0.7 cm diameter (Econo column, Bio-Rad, Hercules, CA), and the column material selected for this purpose was Superose 6 prep grade gel filtration (Amersham-Biosciences, part of GE Healthcare, Piscataway, NJ), which can achieve high-resolution separations across an exceptionally broad molecular weight range. The bed volume of the column was 17.5 mL, and the void volume was 6.0 mL. The composition of the elution buffer was 30 mM HEPES (pH 7.4) containing 150 mM NaCl, 10% glycerol, and 0.5% NP-40. Elution rate was 4.56 mL/h. The column was standardized with the mixture of protein markers containing keyhole limpet hemocyanin (KLH; MW 8.5 million Da), blue dextran (2 million Da), β-amylase (200 kDa), BSA (66 kDa), and cytochrome C (12.4 kDa). The fractions were collected in 500 µL microfuge tubes in a fraction collector. The elution of the markers detected in 280 nm was plotted against the log of the molecular weight of the standard proteins. From this standard elution pattern, the size of the F/P protein network was estimated to be between 2 and 6 million. In a preequilibrated column, we loaded 150 µL (∼3 mg) protein onto the column, and the proteins were separated into 40 tubes, each containing approximately 500 µL column eluent. All the column fractions were stored at −20°C. From each column fraction, 25 µL was taken for analysis by Western blotting with various antibodies. Elution analysis of fractions 8 to 24 were performed in 3 premade gradient SDS-PAGE gels (4%–20%). The proteins were transferred to PVDF membranes. The membranes were blocked with BSA for detection of p-Tyr, and for detection of F/P and other proteins, blocking was carried out with 5% milk, and Western blotting was carried out as described earlier.

### Fluid-phase measurements

MBP values were determined using a double antibody competitive radioimmunoassay [55]. The sensitivities for MBP were less than 1 ng/ml and 2 ng/ml, respectively. The result was adjusted for dilution.

### Statistical analysis

Data are presented as mean ± standard deviation (SD). Data were compared using the two-way analysis of variance (ANOVA) test or independent sample *t* test. *P* values less than 0.05 was considered statistically significant and were derived from 2-tailed statistical test. All statistical treatment was performed using SPSS 13.0 software.

## SUPPLEMENTARY MATERIALS FIGURES


