# Supplementary material for: Lyn mediates FIP1L1-PDGFRA signal pathway facilitating IL-5RA intracellular signal through FIP1L1-PDGFRA/JAK2/Lyn/Akt network complex in CEL
Source: Oncotarget. 2016 Aug 19;8(39):64984–98. doi: 10.18632/oncotarget.11401 (PMC5630306; doi:10.18632/oncotarget.11401)
Supplement: Supplementary file 1 [file oncotarget-08-64984-s001.pdf]

Lyn mediates FIP1L1-PDGFRA signal pathway facilitating IL-5RA intracellular signal through FIP1L1-PDGFRA/JAK2/Lyn/Akt network complex in CEL

Supplementary Materials

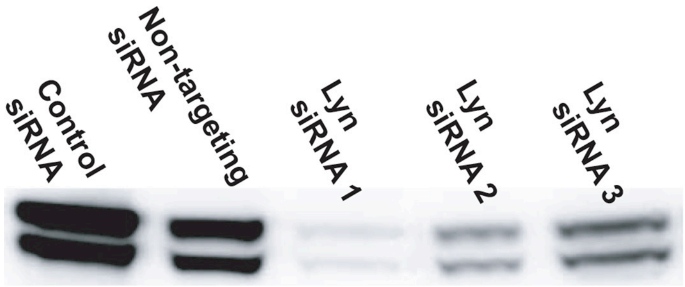

Supplementary Figure S1: Lyn knockdown in EOL-1 by different Lyn siRNAs. EOL-1 cells were transfected with different Lyn siRNAs using lentiviral vector. Whole cell lysates were prepared and subjected to Western blotting. The results showed that the Lyn protein expression was reduced up to about 95% when EOL-1 cells transfected with Lyn siRNA1. Therefore, Lyn siRNA1 was used in the following experiments for Lyn knockdown.

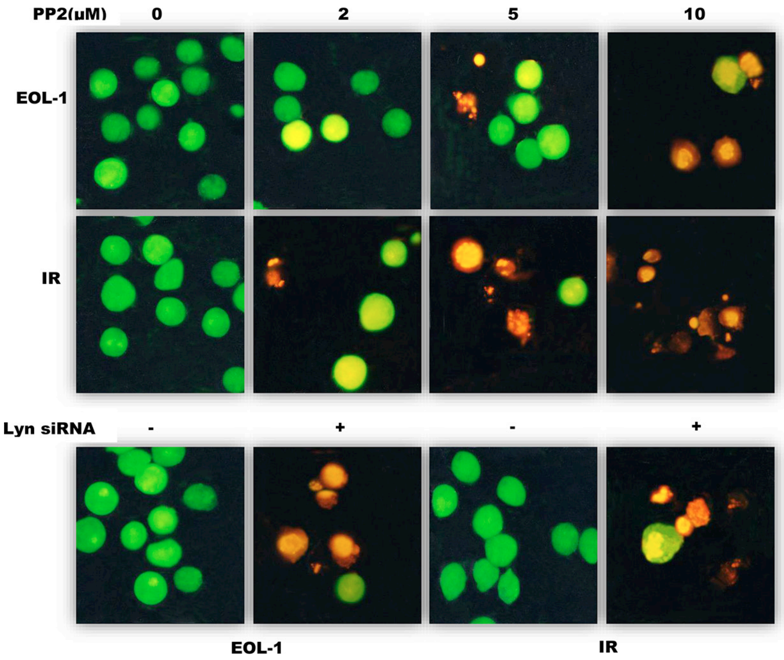

Supplementary Figure S2: Lyn inhibition or knockdown by PP2 or Lyn siRNA induced cellular apoptosis in EOL-1 and IR cells. EoL-1 or IR cells were treated with various concentration of PP2 or transfected with Lyn siRNA. Lyn inhibition increased cellular apoptosis based on the morphological identification when the cells stained with AO/EB and visualized by fluorescence microscopy [Viable cells exhibit a homogeneous green stain; apoptotic cells are orange-red and show evidence of cell shrinkage, nuclear condensation].

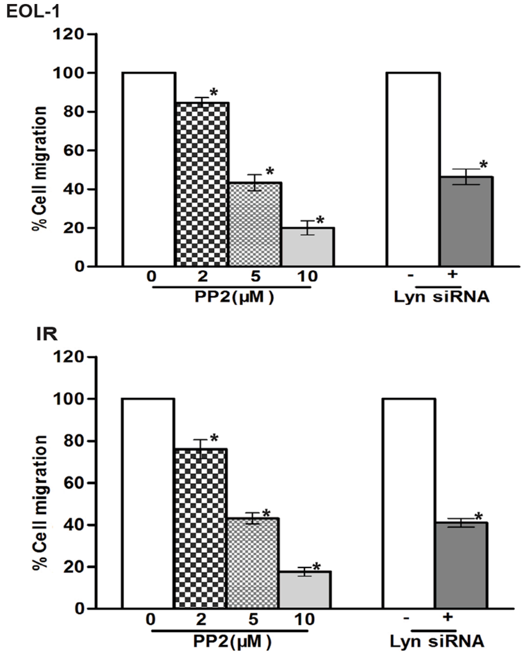

Supplementary Figure S3: Lyn inhibition blocked IL-5-induced migration of EOL-1 and IR cells. The EOL-1 or IR cells treatment with various concentrations of PP2 or transfected with LYN siRNA. Percentage of migrated cells stimulated by IL-5 was calculated. **P* < 0.05, compared to untreated group or control siRNA.

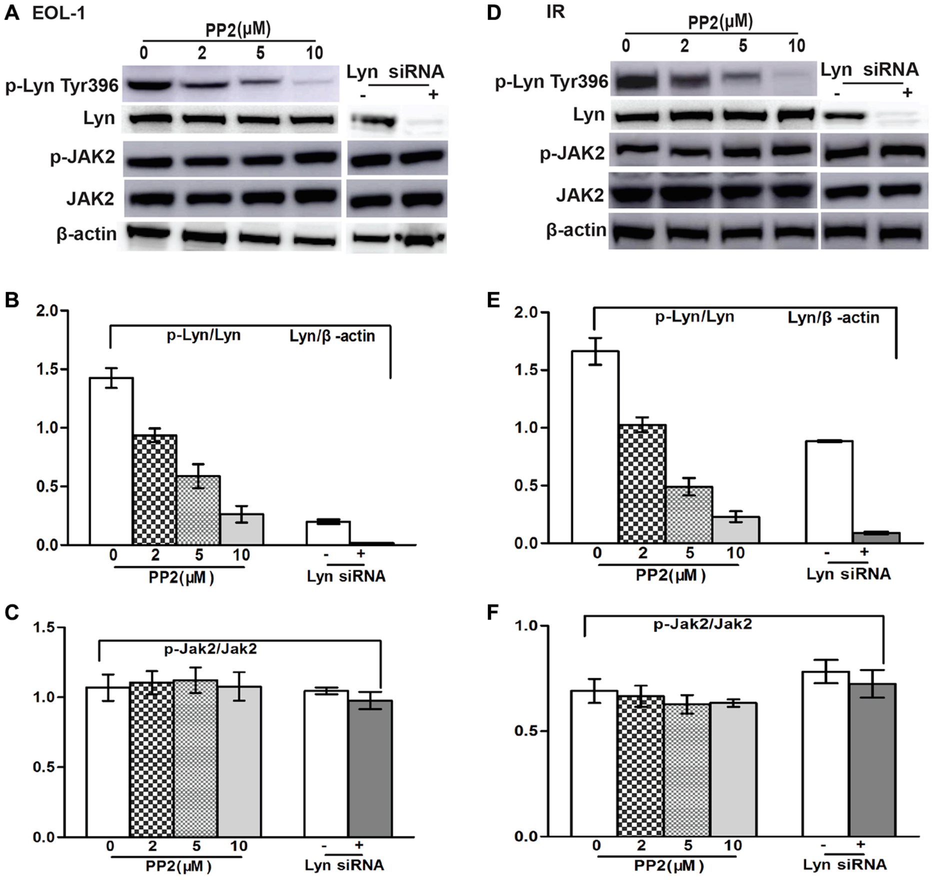

Supplementary Figure S4: Lyn inhibition did not suppress the activation of JAK2 in EOL-1 and IR cells. Cells were treated with various concentration of PP2 or transfected with Lyn siRNA. (**A**) Representative gel images. (**B–C**) Quantification of A. (**D**) Representative gel images. (**E–F**) Quantification of D.

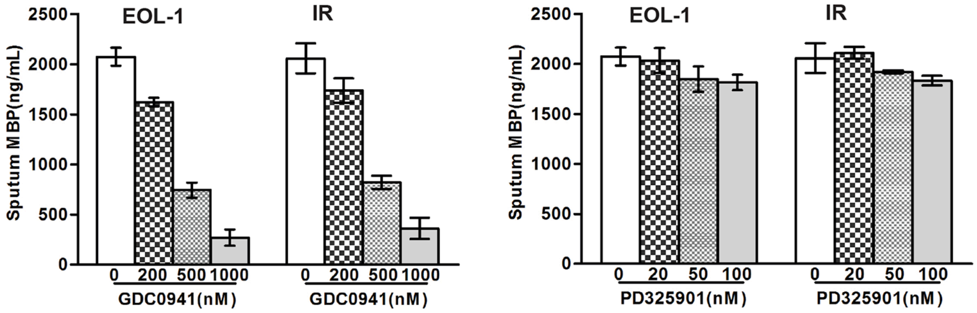

Supplementary Figure S5: PI3Ki, but not MEKi blocked IL-5-induced MBP release in EOL-1 and IR cells. Cells were treated with various concentration of GDC0941[PI3Ki] or PD 325901[MEKi]. MBP values were determined using a double antibody competitive radioimmunoassay.

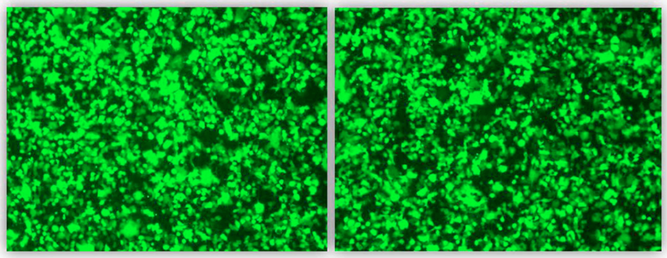

Supplementary Figure S6: Co-expression of IL-5RA and F/P to CD34+ cells using Lentiviral virus vector. Lentiviral DNA constructs were used co-expressing enhanced green fluorescent protein (eGFP) and either IL-5RA[left] or/and FIP1L1-PDGFRA[right], fusing the NH2-terminal 233 amino acids of FIP1L1 to the COOH-terminal 523 amino acids of PDGFRA. The transduction efficacy was proved > 90%.
